# Growth of MIN-6 Cells on Salmon Fibrinogen Scaffold Improves Insulin Secretion

**DOI:** 10.3390/pharmaceutics14050941

**Published:** 2022-04-26

**Authors:** Ivo Laidmäe, Alar Aints, Raivo Uibo

**Affiliations:** 1 Department of Immunology, Institute of Biomedicine and Translational Medicine, Faculty of Medicine, University of Tartu, Ravila 19, 50411 Tartu, Estonia; alar.aints@ut.ee (A.A.); raivo.uibo@ut.ee (R.U.); 2 Institute of Pharmacy, Faculty of Medicine, University of Tartu, Nooruse 1, 50411 Tartu, Estonia; 3 Estonian Academy of Sciences, Kohtu 6, 10130 Tallinn, Estonia

**Keywords:** 3D cell culturing, insulin secretion, electrospinning, salmon fibrinogen, pancreatic islet β-cells

## Abstract

The incidence of type I diabetes has been increasing worldwide at an annual rate of approximately 3%. One of the strategies to treat type I diabetes is islet transplantation, in which damaged β-cells are replaced with new islets. To improve β-cells’ expansion and pseudoislet formation, studies are focusing on using extracellular-matrix-resembling substrates. We evaluated the potential of salmon fibrinogen and chitosan electrospun scaffold as cell substrate for cultivating MIN-6 cells. The morphology of cells, insulin secretion and gene expression was evaluated and compared with other substrates (nanofibrous scaffold, microporous scaffold and tissue culture polystyrene). We found that all tested 3D conditions favored the pseudoislet formation of MIN-6 cells. The insulin secretion of MIN-6 cells after stimulation with high-glucose media shows approximately a 9-fold increase compared to the control group when a fibrinogen/chitosan-based electrospun scaffold was used for cultivation. The differences in insulin secretion were corroborated by differences in gene expression. The differences in insulin secretion could probably be attributed to the differences in the mechanical and/or chemical nature of the tested substrates.

## 1. Introduction

According to a World Health Organization report from 2016, there are over 400 million people in the world with diabetes [1]. The prevalence of diabetes is rising. In the case of type I diabetes, roughly a 3% increase has been reported each year [2]. Type I diabetes patients and the majority of type II diabetes patients suffer from partial or almost complete loss of pancreatic β-cells.

Islet transplantation, in which damaged β-cells are replaced with new islets, has been considered a promising strategy in treating type 1 diabetes [3,4]. Three-dimensional scaffolds have been recognized as useful substrates for generating islet-like structures [5]. It is well understood that the functionality of β-cells is improved when the cells form a spheroid-like (pseudoislet) structure. The formation of pseudoislets is beneficial, especially in terms of stimulus-induced insulin secretion and insulin content [6].

Most β-cell studies use rodent pancreatic cell lines. Murine cell lines (e.g., MIN-6, NIT-1, βTC) and rat cell lines (e.g., RIN, INS-1) are widely used because of their immortality and readiness to secrete insulin after glucose stimulus [7,8,9]. Among these, a mouse pancreatic β-cell line MIN-6 is widely used as a good model to study in vitro β-cell function and transplantation.

Methods for pseudoislet formation include co-culturing with endothelial cells [5] and the use of cryogels [10] and hydrogels [11] as culture support substrates. The use of hydrogels or other extracellular matrix (ECM)-resembling structures together with β-cells has shown great potential for use in transplantation.

Nanofibrous scaffolds, produced by electrospinning, are shown to be suitable cell growth substrates [12]. They are widely studied in various tissue regeneration settings e.g., cardiac tissue engineering [13], bone tissue engineering [14], and vascular tissue engineering [15]. They are also utilized in in vitro β-cell studies, but only a few authors have explored nanofibrous scaffolds as substrates for MIN-6 cells. In summary, a polycaprolactone nanofibrous scaffold coated with a β-cell membrane was studied. Coating synthetic nanostructures with natural cell membranes (cell membrane cloaking technique) gives nanostructures unique cell surface antigens and functions. When such nanofiber scaffolds were used for MIN-6 cell cultures, glucose-dependent insulin secretion significantly increased compared to cells cultured in regular, unmodified nanofiber scaffolds [16]. Another study by Auvro R Mridha et al. used a hybrid device that contained MIN-6 cells enclosed in a polycaprolactone scaffold. They showed that such a subcutaneously implanted hybrid construct normalized the blood glucose of diabetic mice for 2 months [17].

A far greater number of studies have used nanofibrous scaffolds to improve insulin-producing cells’ generation from pluripotent stem cells (reviewed in [18]).

Three-dimensional (3D) fibrous scaffolds are morphologically similar to natural extracellular matrix (ECM) by having a multi-fibrillar network [19]. Production methods (e.g., electrospinning) of nanofibers are flexible enough to allow the use of different chemistries and the design of morphological features into the scaffold (e.g., alignment of fibers, mechanical anisotropy of substrate) [20,21]. Chemical and mechanical cues are crucial for determining the cell fate and can even override each other [22].

Both synthetic and natural materials and their blends are used to produce scaffolds. Polycaprolactone, polylactic acid, polyethylene glycol, poly (lactic-co-glycolic acid), polyurethane and polyaniline are some examples of synthetic materials, and silk protein, chitosan, collagen, gelatin, fibrinogen are commonly used natural materials [23].

We showed earlier that a combination of salmon fibrinogen and chitosan in electrospun scaffolds could serve as a valuable tool for tissue engineering. Wound healing was improved by using fibrinogen/chitosan scaffold compared to no treatment [24]. In addition, fibrin contains sites for cellular binding and promoting good tissue development [25]. Fibrin plays a role in blood clotting, cellular adhesion to a matrix, inflammation and wound healing. It is mediated by interactions between specific binding sites on fibrin or fibrinogen and cell receptors [26].

Salmon-derived fibrin gels support the growth and tubulogenesis of human umbilical vein endothelial cells in vitro [27], and neuronal growth in vitro and in vivo [28,29] is superior to human fibrin gels.

In this study, we evaluated the potential of an electrospun scaffold containing salmon fibrinogen and chitosan for cultivating MIN-6 cells regarding insulin secretion and gene expression, and compared it with other nanofibrous and microporous substrates.

## 2. Materials and Methods

### 2.1. Electrospinning of Chitosan and Fibrinogen/Chitosan Scaffolds

For chitosan scaffold (CS) production, 14 mg/mL chitosan solution (Chitosan 90/100, DDA 90%, batch 212-080119-01, Heppe Medical Chitosan GmbH, Halle, Germany) was prepared using 90% (*v*/*v*) aqueous 1,1,1,3,3,3-hexafluropropan-2-ol (HFP, Lot AS486635, Apollo Scientific, Cheshire, UK) as a solvent. Dissolution was complete after overnight stirring with magnetic stirrer. 

Fibrinogen/chitosan scaffold (FCS) was prepared as described elsewhere [24].

Briefly, salmon fibrinogen (lot# 1513, Sea Run Holdings Inc., Freeport, ME, USA) was dissolved in a mixed solution (ratio 90:10 (*v*/*v*)) of HFP and trifluoroacetic acid (TFA, Lot BCBC3517V, Sigma-Aldrich Laborchemikalien GmbH, Seelze, Germany) at a concentration of 125 mg/mL for 1 h. Chitosan solution was prepared by overnight dissolving in concentrated TFA at concentration of 40 mg/mL. Before electrospinning, chitosan and salmon fibrinogen solutions were mixed (1:1 (*v*/*v*)) for 30 min with a magnetic stirrer. 

For electrospinning, a 10 mL syringe was filled either with chitosan in aqueous HFP or fibrinogen/chitosan in HFP/TFA solution and was equipped with a 25-gauge blunt needle. The syringe was fixed to a syringe pump in robotized electrospinning system (ESR-200R, eS-robot^®^, NanoNC, Seoul, Korea). A grounded plate covered with aluminum foil was used as the fiber collection target.

The following conditions were used for chitosan electrospinning: voltage 18 kV, distance to collector 12.5 cm, solution feeding rate 4 mL/h. For fibrinogen/chitosan electrospinning 15 kV, 15 cm and 0.7 mL/h were used accordingly.

One FCS and one CS scaffold with approximate total surface areas of 100 cm^2^ were electrospun. These scaffolds were used throughout the study.

### 2.2. Scaffold Neutralization and Sterilization

To maintain the 3D fibrous structure of the fibrinogen/chitosan scaffold, neutralization of electrospun matrices was necessary, and this was carried out as described elsewhere [24]. Briefly, a scaffold with suitable size (1.5 × 1.5 cm) was incubated in saturated (prepared as 5 M) Na_2_CO_3_ (Reagent, Donetsk, Russian Federation) aqueous solution for 1 h. Na_2_CO_3_ solution was removed, and the scaffold was washed until neutral pH was reached with deionized water (dH_2_O). The sterilization of scaffolds was carried out with 70% (*v/v*) ethanol (incubation for 1 h in a laminar flow hood) followed by washing with sterile phosphate buffered saline (3 × 5 min with PBS, pH 7.4).

### 2.3. Scanning Electron Microscopy and Image Analysis

The morphology of electrospun products was evaluated by a scanning electron microscope (Zeiss EVO^®^15 MA, Oberkochen, Germany) at the Institute of Ecology and Earth Sciences, University of Tartu. Samples were mounted on a sample holder with adhesive carbon tape and sputter coated with 3 nm gold layer in argon atmosphere. ImageJ (version 1.52b) software (National Institutes of Health, Bethesda, MD, USA) was used to analyze scaffold morphology. The diameters of 100 randomly selected fibers were measured from the SEM images [30].

### 2.4. MIN-6 Cell Culture

Equal amounts (1.5 × 10^5^) of MIN-6 cells (kindly donated by Professor Timo Otonkoski, University of Helsinki) were seeded either on (1) the standard tissue culture polystyrene plate (“TCP”, 24 well, Corning), (2) the 3D polystyrene scaffold (“A”, Alvetex^®^, prod no AVP002, Lot: 5REN001), (3) the sterilized chitosan scaffold (“CS”) or (4) the neutralized and sterilized fibrinogen/chitosan scaffold (“FCS”). Scaffolds were mounted in standard 24-well TCP using CellCrown inserts (CellCrown 24, Scaffdex Oy, Tampere, Finland).

MIN-6 cells were cultured in Dulbecco’s modified Eagle’s medium containing GlutaMax, 4.5 g/L d-glucose, and 25 mM HEPES; 15% fetal bovine serum and 100 U/mL penicillin/streptomycin (Gibco Life Technologies, Paisley, UK), with 70 µM β-mercaptoethanol (Ferak Laborat GmbH, Berlin (West), Germany). HEPES at 37 °C and 5% CO_2_ atmosphere. The media were changed every 2–3 days.

### 2.5. Phase Contrast and Stereomicroscopy

The cell morphology of TCP and scaffolds was periodically monitored during culturing with inverted phase contrast microscope Olympus CK40 (Olympus Corporation, Tokyo, Japan) and stereomicroscope (Nikon SMZ645, Nikon Corporation, Tokyo, Japan).

### 2.6. Cell Proliferation Assay

MIN-6 proliferation was evaluated at day 10 after seeding by the MTS assay (CellTiter 96 Aqueous One Solution Cell Proliferation Assay, Promega Corporation, Madison, WI, USA). After removing the culture medium, the cells were washed with PBS (pH 7.4). After this, 1000 μL serum-free DMEM medium and 200 μL MTS solution were added to sample wells and incubated for 1.5 h at 37 °C and 5% CO_2_ atmosphere. Obtained reaction products were collected and put into 96-well plates (150 μL per well). The optical density of the plate wells was measured using a microplate reader (Victor X, Perkin Elmer, Waltham, MA, USA) at 490 nm. Cells were washed with PBS (3 × 5 min) and cultured further with regular medium. 

### 2.7. Insulin Secretion Assay

Insulin secretion from MIN-6 cells in response to glucose (25 mM) stimulation was measured at day 10 with Mercodia Ultrasensitive Mouse Insulin ELISA kit (Mercodia, Uppsala, Sweden). The culture medium was removed, and cells were washed with PBS (2 × 5 min). Further, 1 mL serum-free DMEM with 1 mM glucose was added and cells were incubated for 1 h at 37 °C and 5% CO_2_ atmosphere (equilibration period). Then the culture medium was removed. Then, 1 mL serum-free DMEM with 25 mM glucose (stimulated conditions) was added, and cells were incubated for 1 h. After this, culture media were collected and stored at −80 °C for later insulin measurement. Cells were washed with PBS (2 × 5 min) and cultured with regular media.

Insulin measurement in collected culture supernatant samples was carried out according to Mouse Insulin ELISA kit instructions and the optical density at 450 nm was recorded with a Victor X multilabel plate reader. A 1:50 dilution of supernatant samples was needed to fit the standard curve range.

### 2.8. RNA Extraction

Total RNA extraction from MIN-6 cells in all study groups was conducted after 10 days of cultivation using the next protocol. Briefly, 600 μL cetyltrimethylammonium bromide (CTAB, AppliChem GmbH, Darmstadt, Germany) buffer containing 1% β-mercaptoethanol (Ferak Laborat GmbH, Berlin (West), Germany) was added to cells on TCP or cell-containing scaffolds (scaffolds were removed from CellCrown inserts and transferred to 1.5 mL microcentrifuge tubes). After short mixing, 600 μL chloroform was added, and tubes were mixed well with vortex. Then, centrifugation at full speed (16,500× *g*, Eppendorf 5418, Eppendorf AG, Hamburg, Germany) was carried out for 2 min. The upper phase was collected, to which an equal amount of isopropanol was added and mixed again, followed by centrifugation for 15 min at full speed, and the supernatant was discarded. Next, 600 μL of 70% ethanol was added to wash the pellet, followed by centrifugation at maximum speed for 5 min. Air-dried pellet was dissolved in RNase free water and was kept at −80 °C. The quality of isolated RNA was verified by agarose gel electrophoresis and absorbance reads (260, 280 and 230 nm) using a NanoDrop (Thermo Fisher Scientific Inc., Waltham, MA, USA) spectrometer.

### 2.9. cDNA Synthesis and Gene Expression Analysis

Reverse transcription was carried out with random hexamer primers, according to manufacturer’s instructions using SuperScript III reverse transcriptase (Invitrogen, Carlsbad, CA, USA). Real-time PCR for genes of interest (*Ins1*, *Ins2*, *Itga6*, *Itgb1*, *Tspan7*, *Cldn7*, *Ceacam1*, *Cdh1*, *Scg5*, *Scg3*, *Chgb*) was performed under standard conditions on ABI Prism 7000 Sequence Detection System (Applied Biosystems, Foster City, CA, USA) using Maxima SYBR green/ROX qPCR mastermix (Thermo Fischer Scientific Baltics OAS, Vilnius, Lithuania). Samples were run in two parallel experiments. Data were normalized to β-actin as a housekeeping gene (*Actb*). Relative expression levels were expressed as –ΔΔCt. The primers for PCR were designed to bind in separate exons to avoid interference from genomic DNA, and they were synthesized by TAG Copenhagen (Copenhagen, Denmark). Primers for the target genes and housekeeping *Actb* are listed in Table 1.

### 2.10. Statistical Analysis

Comparisons were performed using the Student’s *t*-test between two groups with Excel for Microsoft 365. The results are presented as the mean ± SD. *p* < 0.05 is considered statistically significant.

## 3. Results and Discussion

### 3.1. Electrospinning of Chitosan and Fibrinogen/Chitosan Scaffolds

**Figure 1:**
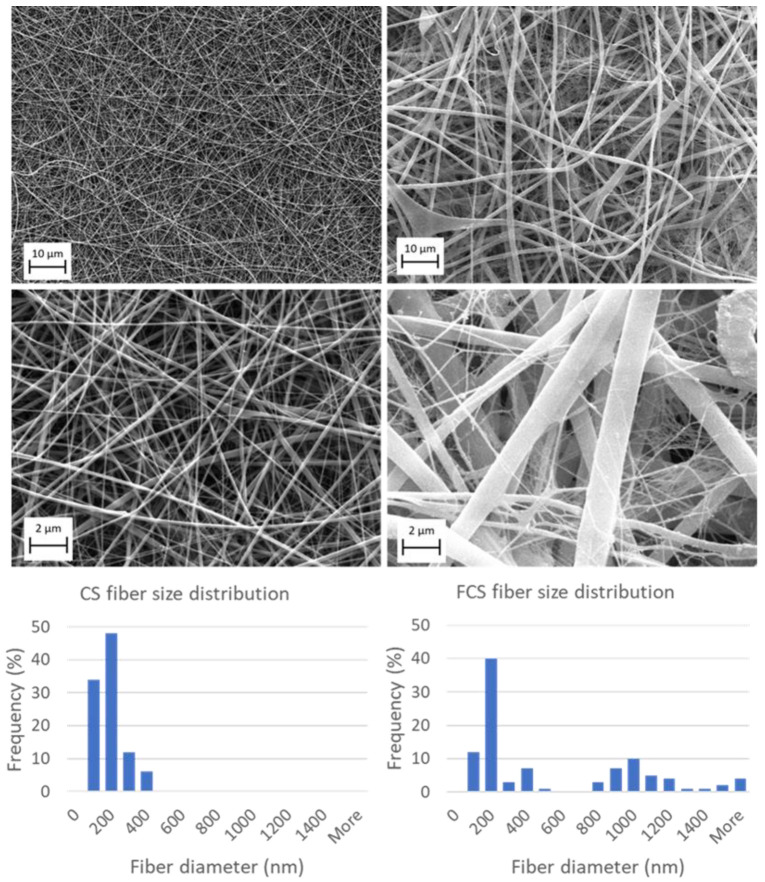
SEM and fiber diameter distributions of chitosan (CS, left column) and fibrinogen/chitosan (FCS, right column) scaffolds.

The SEM image of CS is shown in Figure 1. The obtained fibers had smooth and uniform surfaces with no bead-like structures. Electrospinning allowed chitosan fibers to be produced with mean diameters (±SD) of 144.1 ± 70.6 nm. The size distribution was uniform.

The fibrinogen/chitosan scaffold structure (Figure 1) is more heterogeneous than that of CS and contains some beads and inconsistencies. Size distribution follows a bimodal pattern (Figure 1) involving small fibers (ranging approximately from 75–600 nm) and large fibers (ranging from 800 nm to 3.7 µm). Table 2 summarizes the key morphological parameters for electrospun scaffolds.

### 3.2. Cell Cluster Morphology

Cell cluster morphology was regularly evaluated with inverted phase-contrast and stereomicroscopy during culture medium changes and was imaged on days 18 and 37. MIN-6 cells already formed islet-like structures (pseudoislets) on 3D scaffolds (A, CS and FCS) at the early phase (days 5–10) of cultivation (Figure S1). Some minimal aggregation could also be seen on the TCP plate. The tendency of MIN-6 cells to aggregate and form loose and irregular aggregates on nontreated dishes was also shown earlier [31]. The formation of aggregates is demonstrated to be more evident when using low attachment microenvironment (e.g., low attachment TCP or suspension cultivation) [32,33]. Additionally, 3D substrates (especially hydrogels) or constrained spaces (microwell culturing) are known techniques for the cultivation of MIN-6 cells to encourage pseudoislet formation.

**Figure 2:**
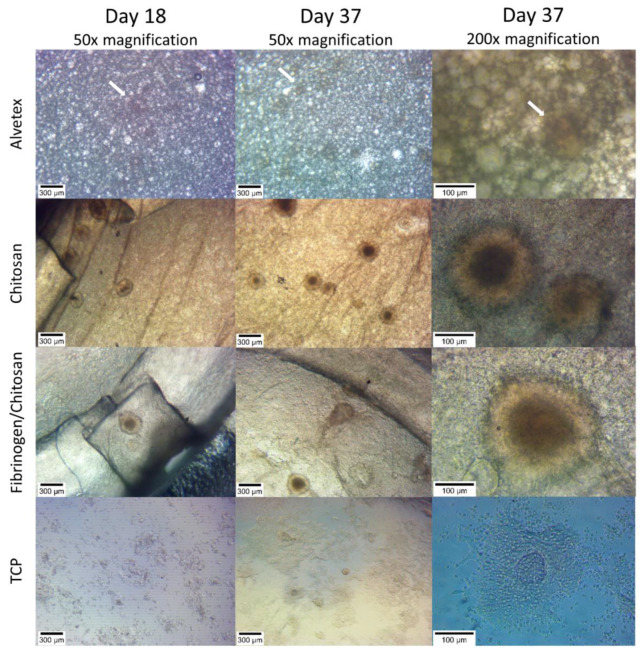
Formation of pseudoislets on 3D scaffolds. White arrows indicate poorly visible pseudoislet structures in Alvetex scaffold.

**Figure 3:**
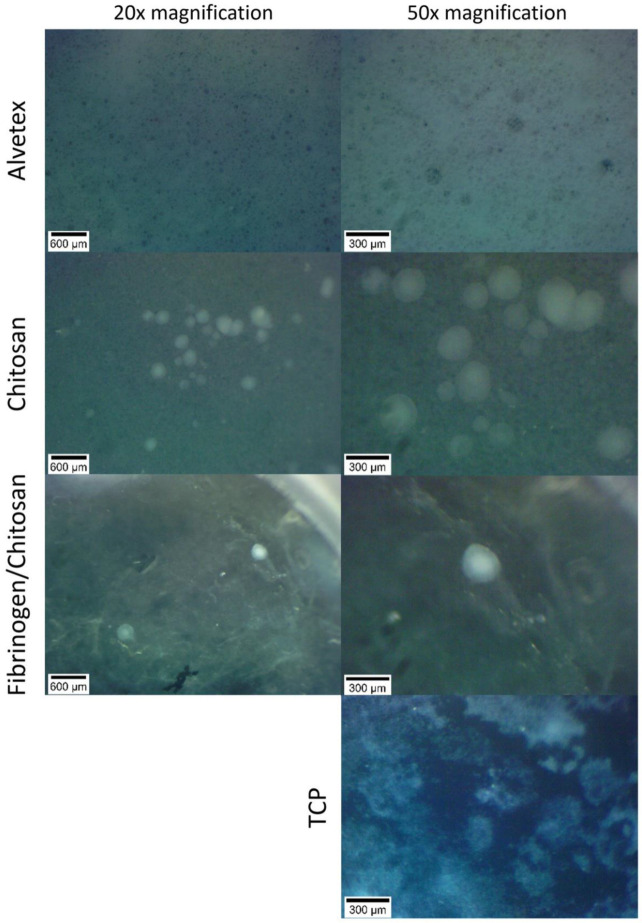
Stereomicroscope images of pseudoislets on 3D scaffolds at day 37.

Pseudoislet formation revealed by phase-contrast microscopy and stereomicroscopy is shown in Figure 2 and Figure 3, respectively. The size of the pseudoislets on FCS and CS scaffolds reached up to 300 µm. The formation of pseudoislets is also seen on Alvetex scaffold, but the structures are not as clear. All of the pictures are imaged using an inverted microscope from the underside of the scaffold material with cells growing on top of it. This, and the thickness of material, may therefore limit the imaging clarity of the cell aggregates as seen in case of Alvetex.

Using a stereomicroscope, we were able to image the pseudoislets directly on top of the scaffold. In the case of the Alvetex scaffold, no visible structures could be identified. According to the manufacturer’s website, Alvetex scaffold voids have an average diameter of 40 µm with interconnects of approximately 13 µm in diameter. It is probable that the pseudoislets are completely embedded in the microporous structure of the surrounding material and cannot be visualized by the current method.

### 3.3. Insulin Secretion

**Figure 4:**
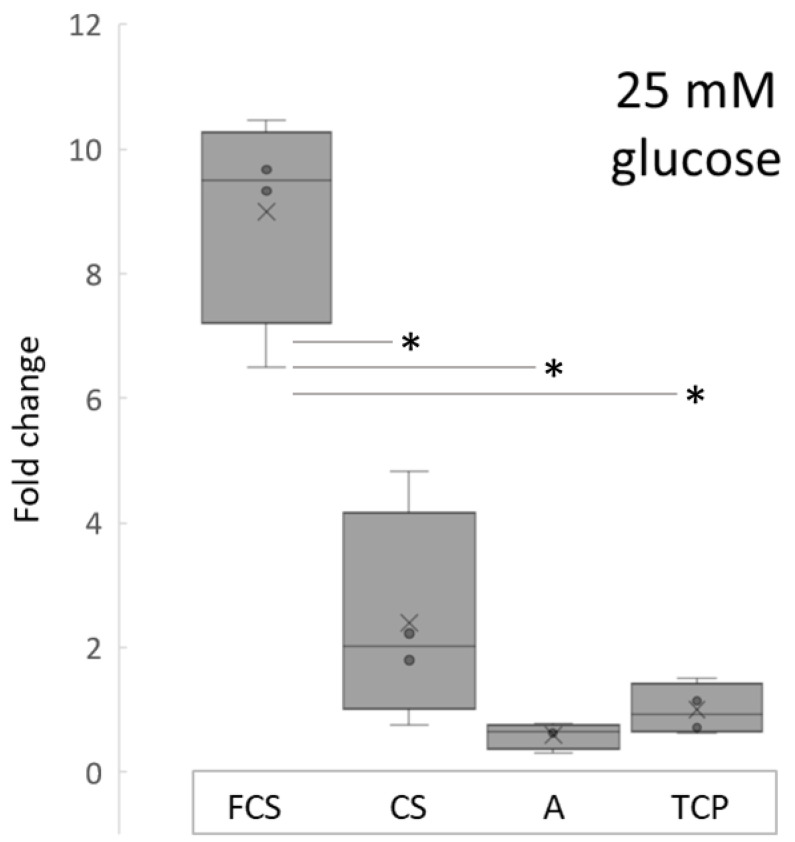
Normalized induced insulin secretion relative to TCP in supernatants of MIN-6 cells cultured on different scaffolds, after 25 mM glucose challenge. Insulin values were normalized to the cell counts in each group. * *p* ≤ 0.005.

The insulin secretion of MIN-6 cells was measured at day 10 after incubating cells in 25 mM glucose conditions. Insulin values (Figure 4) were normalized to the cell counts as detected with an MTS proliferation assay. The lowest insulin concentration was detected in Alvetex group (1.04 ± 0.36 µg/L), followed by TCP (1.76 ± 0.72 µg/L), CS group (4.22 ± 3.04 µg/L) and FCS group (15.81 ± 3.05 µg/L). Insulin secretion was almost 9 times higher in FCS group compared to TCP, and 4 times higher compared to CS group, and was significantly different when compared to other study group values.

Although the mean value of insulin concentration in the chitosan (CS) group was higher compared to the Alvetex (A) and TCP group, it was not statistically significant. Similar results can be seen in studies conducted using hydrogel substrates. MIN-6 cells cultivated in 3D hydrogels show a higher insulin secretion compared to monolayers [11]. Moreover, authors found that cells grown on a mechanically softer substrate with viscoelastic properties secrete more insulin. Pancreatic β-cells’ viability and functioning are affected by the local mechanical microenvironment.

Substrate stiffness and its impact to β-cell function has been specifically studied by Crystal E. Nyitray and colleagues. They showed that three-dimensional primary mouse islet-derived and MIN-6 β-cell clusters increased insulin mRNA expression when grown on soft substrates (0.1 kPa). These scaffolds also increased the glucose sensitivity of MIN-6 β-cell clusters. This was demonstrated by the improved glucose stimulation index [34].

We tested the viscoelastic properties of the fibrinogen/chitosan electrospun material in our previous study and found that the shear modulus of the hydrated material is in the order of several hundred pascals (Pa) [24], which is lower than the reported values for chitosan-based meshes (in range of few MPa-s [35,36]. The reported value of Young’s modulus for Alvetex is 77 kPa [37] and, for the tissue culture polystyrene (TCP), it is in the range of few GPa-s [38]. This could partly explain, apart from chemical differences, the higher insulin values in the FCS group compared to the pure chitosan scaffold group. To draw solid conclusions with regard to scaffold mechanical properties’ impact on insulin secretion, further studies focusing on rheological properties of scaffolds in the same experimental settings are needed.

In accordance with our results, it has been shown that functionality (e.g., insulin secretion) of MIN-6 cells is increased not only by pseudoislet formation, but it is also dependent on the stiffness, and adhesion ligands of the cellular microenvironment [11,16]. Our results show that all 3D microenvironments (FCS, CS and A) encouraged the formation of pseudoislets, but insulin secretion was different. This could be due to differences in mechanical properties or chemical nature of the materials used in this study. The fibrinogen used in the current study contains fibronectin as a copurifying substance. This might potentially contribute to better adhesion by binding to cell surface integrins [39].

### 3.4. Gene Expression

**Figure 5:**
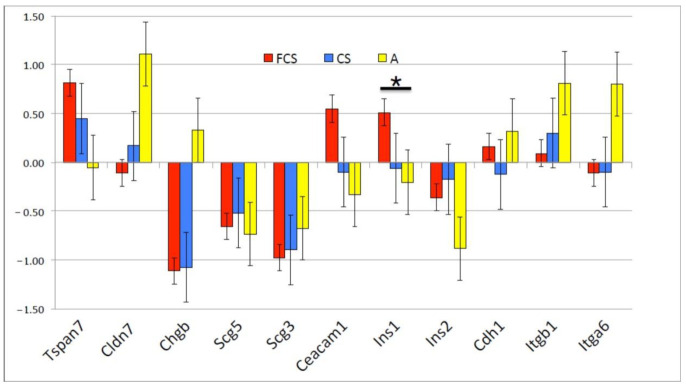
Gene expression differences after 10 days of culture (*n* = 2) as measured by rt-PCR and displayed as –ΔΔCt (±SD group average). * *p* < 0.05.

The differences in insulin secretion were corroborated by differences in gene expression. Relative mRNA expression levels were measured using real-time PCR method, using *Actin B* gene as a reference standard. The differences are shown relative to control cultures (i.e., cells growing on tissue culture plates without a 3D matrix), as the reciprocal of the cycle time difference (−ΔΔCt) (Figure 5). We measured mRNA levels of genes belonging to three groups of genes: Adhesion-related—*Tetraspanin 7 (Tspan7), Claudin 7 (Cldn7), Cadherin 1 (Cdh1), Integrin beta 1 (Itgb1), Integrin alpha 6 (Itga6), and Carcinoembryonic antigen-related cell adhesion molecule 1 (Ceacam1);* Secretion-related—*Chromogranin B (ChgB), Secretogranin 5 (Scg5), and Secretogranin 3 (Scg3)*; and *Insulin 1 (Ins1), Insulin 2 (Ins2).* The culture on FCS upregulated the expression of *Ins1, Ceacam1 and Tspan7* mRNAs, whereas secretion-related genes’ (*Chgb*, *Scg5* and *Scg3*) mRNA levels were downregulated. We interpret this as a possible post-transcriptional mechanism of secretion regulation in addition to mRNA level regulation. The increase in *Insulin1* mRNA was accompanied by a measurable increase in detectable protein. The culture on chitosan did not change *Insulin* mRNA expression compared to standard 2D culture; on Alvetex, there was a tendency of *Insulin* mRNA downregulation. Overall, the differences in Ct were not large, 0.5–1, which corresponded to a 1.5–2-fold difference at the mRNA level.

## 4. Conclusions

Electrospun FCS is a morphologically heterogeneous (nano- and microfibrous) scaffold resembling extracellular matrix. The mean fiber diameter of an FCS scaffold is 525.9 ± 576.1 nm and size distribution follows a bimodal pattern. In contrast, an electrospun CS scaffold is a homogeneous nanofibrous scaffold with a mean fiber diameter of 144.1 ± 70.6 nm. From our results, it can be concluded that all tested 3D scaffolds (nano- and microfibrous fibrinogen/chitosan scaffold, nanofibrous chitosan scaffold and microporous Alvetex scaffold) support spontaneous MIN-6 cells pseudoislet formation. The size of the pseudoislets on FCS and CS scaffolds reached up to 300 µm by day 37. Gene expression analysis revealed that the culture on FCS upregulated the expression of *Ins1*, *Ceacam1 and Tspan7* mRNAs. The increase in *Insulin1* mRNA was accompanied by measurable increase in detectable protein. Insulin secretion after stimulation in the FCS group showed approximately a 9-fold increase compared to the control and was superior to other tested conditions. This could be probably attributed to the mechanical and/or chemical nature of the FCS scaffold.

## Figures and Tables

**Table 1:** PCR primers used for gene expression analysis.

Genes	Sequences
tspan7_Mm_5′974	TCTGCCTTTCAGCCCACGTC
tspan7_Mm_3′1167	CTGAAGCCTCCCCTACTACATGC
cldn7_Mm_5′612	GCGGGCGACAACATCATCACA
cldn7_Mm_3′784	ATCGTGGCGACAAACATGGCTA
chgb_Mm_5′223	CCCTATCCAAGTCCAGTGTTCCAA
chgb_Mm_3′434	CACTTCTCATTGCCTACCTTCGTC
scg5_Mm_5′260	CTCACCAGGCCATGAATCTTGTTG
scg5_Mm_3′489	ACTGGAATTCTCGGCTGAACTCT
scg3_Mm_5′202	CTCTCCCTTCCCGCACCCAG
scg3_Mm_3′344	CAGTATCCAAGAGCCGGTCCA
ceacam1_Mm_5′529	GCCCTTCCTCCAAGTCACCAAC
ceacam1_Mm_3′730	CGCTGACTGGATTCGAGATTTCACAC
ins1_Mm_5′268	AACCCACCCAGGCTTTTGTCA
ins1_Mm_3′464	ACTGATCCACAATGCCACGCTTC
ins2_Mm_5′139	CCCCACCCAGGCTTTTGTCA
ins2_Mm_3′340	ACTGATCTACAATGCCACGCTTC
actb_Mm_5′367	GCACCACACCTTCTACAATGAGC
actb_Mm_3′558	CTCCGGAGTCCATCACAATGC
cdh1_Mm_5′930	CAGAGTTTACCCAGCCGGTCT
cdh1_Mm_3′1149	ATGTAGGGTAACTCTCTCGGTCCA
itgb1_Mm_5′532	CAGCCAAGTGACATAGAGAATCCCA
itgb1_Mm_3′840	GCCAAAGCCAATGCGGAAGTCT
itga6_Mm_5′1080	AGAGACATGAAGTCCGCGCATC
itga6_Mm_3′1282	ACGAATCGGCTTCACATTACTCC

**Table 2:** Key fiber parameters of electrospun scaffolds.

	CS Scaffold	FCS Scaffold
Mean diameter (nm ± *SD*)	144.1 ± *70.6*	525.9 ± *576.1*
Median diameter (nm)	115.0	192.8
Minimum diameter (nm)	67.4	76.9
Maximum diameter (nm)	391.7	3743.4
Mid-range value (nm ± *SD*)	229.6 ± *229.3*	1910.2 ± *2592.6*

## Data Availability

The data presented in this study are available on request from the corresponding author. The data are not publicly available as they also form part of an ongoing study.

## Supplementary Materials

The following supporting information can be downloaded at: https://www.mdpi.com/article/10.3390/pharmaceutics14050941/s1, Figure S1: Images taken with smartphone camera through the ocular of the phase contrast microscope (50× magnification) showing pseudoislet formation of MIN-6 cells on chitosan (CS, left) and Fibrinogen/chitosan scaffold (FCS, right) at day 8 of culture.

## References

[1] World Health Organization Global Reports on Diabetes 2016. http://www.who.int/diabetes/global-report/en/.

[2] Borchers A.T., Uibo R., Gershwin M.E. (2010). The geoepidemiology of type 1 diabetes. Autoimmun. Rev..

[3] Khan K., Desai C.S. (2019). Islet Transplantation in Children. Curr. Gastroenterol. Rep..

[4] Rickels M.R., Robertson R.P. (2019). Pancreatic Islet Transplantation in Humans: Recent Progress and Future Directions. Endocr. Rev..

[5] Vlahos A.E., Kinney S.M., Kingston B.R., Keshavjee S., Won S.-Y., Martyts A., Chan W.C.W., Sefton M.V. (2020). Endothelialized collagen based pseudo-islets enables tuneable subcutaneous diabetes therapy. Biomaterials.

[6] Aloysious N., Nair P.D. (2014). Enhanced survival and function of islet-like clusters differentiated from adipose stem cells on a three-dimensional natural polymeric scaffold: An in vitro study. Tissue Eng. Part A.

[7] Green A.D., Vasu S., Flatt P.R. (2018). Cellular models for beta-cell function and diabetes gene therapy. Acta Physiol..

[8] Ishihara H., Asano T., Tsukuda K., Katagiri H., Inukai K., Anai M., Kikuchi M., Yazaki Y., Miyazaki J.I., Oka Y. (1993). Pancreatic beta cell line MIN6 exhibits characteristics of glucose metabolism and glucose-stimulated insulin secretion similar to those of normal islets. Diabetologia.

[9] Spelios M.G., Olsen J.A., Kenna L.A., Akirav E.M. (2015). Islet Endothelial Cells Induce Glycosylation and Increase Cell-surface Expression of Integrin β1 in β Cells. J. Biol. Chem..

[10] Velasco-Mallorquí F., Rodríguez-Comas J., Ramón-Azcón J. (2021). Cellulose-based scaffolds enhance pseudoislets formation and functionality. Biofabrication.

[11] Zhang M., Yan S., Xu X., Yu T., Guo Z., Ma M., Zhang Y., Gu Z., Feng Y., Du C. (2021). Three-dimensional cell-culture platform based on hydrogel with tunable microenvironmental properties to improve insulin-secreting function of MIN6 cells. Biomaterials.

[12] Reddy V.S., Tian Y., Zhang C., Ye Z., Roy K., Chinnappan A., Ramakrishna S., Liu W., Ghosh R. (2021). A Review on Electrospun Nanofibers Based Advanced Applications: From Health Care to Energy Devices. Polymers.

[13] Suh T.C., Amanah A.Y., Gluck J.M. (2020). Electrospun Scaffolds and Induced Pluripotent Stem Cell-Derived Cardiomyocytes for Cardiac Tissue Engineering Applications. Bioengineering.

[14] Yao Q., Cosme J.G.L., Xu T., Miszuk J.M., Picciani P.H.S., Fong H., Sun H. (2017). Three dimensional electrospun PCL/PLA blend nanofibrous scaffolds with significantly improved stem cells osteogenic differentiation and cranial bone formation. Biomaterials.

[15] El-Ghazali S., Khatri M., Hussain N., Khatri Z., Yamamoto T., Kim S.H., Kobayashi S., Kim I.S. (2021). Characterization and biocompatibility evaluation of artificial blood vessels prepared from pristine poly (Ethylene-glycol-co-1,4-cyclohexane dimethylene-co-isosorbide terephthalate), poly (1, 4 cyclohexane di-methylene-co-isosorbide terephthalate) nanofibers and their blended composition. Mater. Today Commun..

[16] Chen W., Zhang Q., Luk B.T., Fang R.H., Liu Y., Gao W., Zhang L. (2016). Coating nanofiber scaffolds with beta cell membrane to promote cell proliferation and function. Nanoscale.

[17] Mridha A.R., Dargaville T.R., Dalton P.D., Carroll L., Morris M.B., Vaithilingam V., Tuch B.E. (2022). Prevascularized Retrievable Hybrid Implant to Enhance Function of Subcutaneous Encapsulated Islets. Tissue Eng. Part A.

[18] Hoveizi E., Tavakol S., Shirian S., Sanamiri K. (2019). Electrospun Nanofibers for Diabetes: Tissue Engineering and Cell-Based Therapies. Curr. Stem Cell Res. Ther..

[19] Xu Y., Shi G., Tang J., Cheng R., Shen X., Gu Y., Wu L., Xi K., Zhao Y., Cui W. (2020). ECM-inspired micro/nanofibers for modulating cell function and tissue generation. Sci. Adv..

[20] Kim J.I., Hwang T.I., Aguilar L.E., Park C.H., Kim C.S. (2016). A Controlled Design of Aligned and Random Nanofibers for 3D Bi-functionalized Nerve Conduits Fabricated via a Novel Electrospinning Set-up. Sci. Rep..

[21] Nieminen H.J., Laidmäe I., Salmi A., Rauhala T., Paulin T., Heinämäki J., Hæggström E. (2018). Ultrasound-enhanced electrospinning. Sci. Rep..

[22] Janmey P.A., Winer J.P., Murray M.E., Wen Q. (2009). The hard life of soft cells. Cell Motil. Cytoskelet..

[23] Bhattarai D.P., Aguilar L.E., Park C.H., Kim C.S. (2018). A Review on Properties of Natural and Synthetic Based Electrospun Fibrous Materials for Bone Tissue Engineering. Membranes.

[24] Laidmäe I., Ērglis K., Cēbers A., Janmey P.A., Uibo R. (2018). Salmon fibrinogen and chitosan scaffold for tissue engineering: In vitro and in vivo evaluation. J. Mater. Sci. Mater. Med..

[25] Uibo R., Laidmäe I., Sawyer E.S., Flanagan L.A., Georges P.C., Winer J.P., Janmey P.A. (2009). Soft materials to treat central nervous system injuries: Evaluation of the suitability of non-mammalian fibrin gels. Biochim. Biophys. Acta.

[26] Laurens N., Koolwijk P., de Maat M.P.M. (2006). Fibrin structure and wound healing. J. Thromb. Haemost..

[27] Sieminski A.L., Gooch K.J. (2004). Salmon fibrin supports an increased number of sprouts and decreased degradation while maintaining sprout length relative to human fibrin in an in vitro angiogenesis model. J. Biomater. Sci. Polym. Ed..

[28] Ju Y.-E., Janmey P.A., McCormick M.E., Sawyer E.S., Flanagan L.A. (2007). Enhanced neurite growth from mammalian neurons in three-dimensional salmon fibrin gels. Biomaterials.

[29] Sharp K.G., Dickson A.R., Marchenko S.A., Yee K.M., Emery P.N., Laidmåe I., Uibo R., Sawyer E.S., Steward O., Flanagan L.A. (2012). Salmon fibrin treatment of spinal cord injury promotes functional recovery and density of serotonergic innervation. Exp. Neurol..

[30] Schindelin J., Arganda-Carreras I., Frise E., Kaynig V., Longair M., Pietzsch T., Preibisch S., Rueden C., Saalfeld S., Schmid B. (2012). Fiji: An open-source platform for biological-image analysis. Nat. Methods.

[31] Yang K.-C., Wu C.-C., Yang S.-H., Chiu C.-C., Sumi S., Lee H.-S. (2013). Investigating the suspension culture on aggregation and function of mouse pancreatic β-cells. J. Biomed. Mater. Res. A.

[32] Zhi Z.-L., Liu B., Jones P.M., Pickup J.C. (2010). Polysaccharide multilayer nanoencapsulation of insulin-producing beta-cells grown as pseudoislets for potential cellular delivery of insulin. Biomacromolecules.

[33] Kelly C., Guo H., McCluskey J.T., Flatt P.R., McClenaghan N.H. (2010). Comparison of insulin release from MIN6 pseudoislets and pancreatic islets of Langerhans reveals importance of homotypic cell interactions. Pancreas.

[34] Nyitray C.E., Chavez M.G., Desai T.A. (2014). Compliant 3D microenvironment improves β-cell cluster insulin expression through mechanosensing and β-catenin signaling. Tissue Eng. Part A.

[35] Gu B.K., Park S.J., Kim M.S., Kang C.M., Kim J.-I., Kim C.-H. (2013). Fabrication of sonicated chitosan nanofiber mat with enlarged porosity for use as hemostatic materials. Carbohydr. Polym..

[36] Tuzlakoglu K., Alves C.M., Mano J.F., Reis R.L. (2004). Production and characterization of chitosan fibers and 3-D fiber mesh scaffolds for tissue engineering applications. Macromol. Biosci..

[37] Asthana A., Kisaalita W.S. (2013). Biophysical microenvironment and 3D culture physiological relevance. Drug Discov. Today.

[38] Yang C., Tibbitt M.W., Basta L., Anseth K.S. (2014). Mechanical memory and dosing influence stem cell fate. Nat. Mater..

[39] Laidmäe I., McCormick M.E., Herod J.L., Pastore J.J., Salum T., Sawyer E.S., Janmey P.A., Uibo R. (2006). Stability, sterility, coagulation, and immunologic studies of salmon coagulation proteins with potential use for mammalian wound healing and cell engineering. Biomaterials.

