# Supplementary material for: Growth of MIN-6 Cells on Salmon Fibrinogen Scaffold Improves Insulin Secretion
Source: Pharmaceutics. 2022 Apr 26;14(5):941. doi: 10.3390/pharmaceutics14050941 (PMC9144899; doi:10.3390/pharmaceutics14050941)
Supplement: Supplementary file 1 [file pharmaceutics-14-00941-s001.zip › pharmaceutics-1684194-supplementary.pdf]

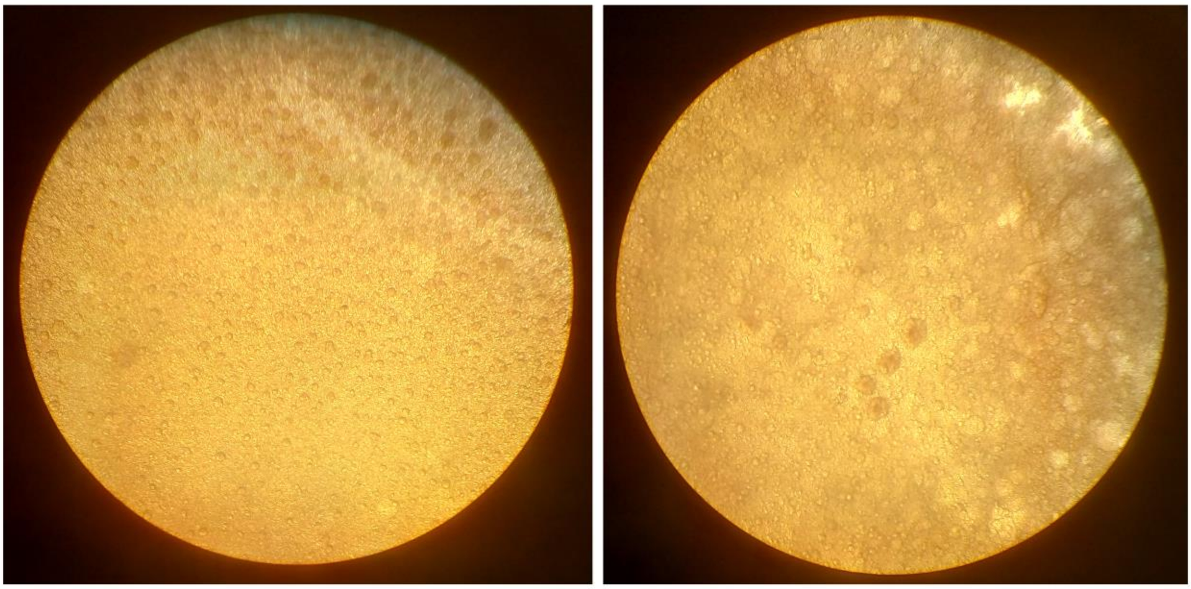

Figure S1: Images taken with smartphone camera through the ocular of the phase contrast microscope (50x magnification) showing pseudoislet formation of MIN-6 cells on chitosan (CS, left) and Fibrinogen/chitosan scaffold (FCS, right) at day 8 of culture.
